# Supplementary material for: In-Situ Sludge Reduction Performance and Mechanism in Sulfidogenic Anoxic–Oxic–Anoxic Membrane Bioreactors
Source: Membranes (Basel). 2022 Sep 8;12(9):865. doi: 10.3390/membranes12090865 (PMC9502630; doi:10.3390/membranes12090865)
Supplement: Supplementary file 1 [file membranes-12-00865-s001.zip › membranes-1840945-supplementary.pdf]

Supplementary Material

In–Situ Sludge Reduction Performance and Mechanism in Sulfidogenic Anoxic–Oxic–Anoxic Membrane Bioreactors

Chengyue Li [1,2,3,†], Tahir Maqbool [1,2,3,†], Hongyu Kang [1,2,3] and Zhenghua Zhang [1,2,3,*]

1 Institute of Environmental Engineering & Nano-Technology, Tsinghua Shenzhen International Graduate School, Tsinghua University, Shenzhen 518055, China
2 Guangdong Provincial Engineering Research Centre for Urban Water Recycling and Environmental Safety, Tsinghua Shenzhen International Graduate School, Tsinghua University, Shenzhen 518055, China
3 School of Environment, Tsinghua University, Beijing 100084, China
* Correspondence: zhenghua.zhang@sz.tsinghua.edu.cn
† These authors contributed equally to this work.

Text S1. PARAFAC modeling

The fluorescence spectrometer (F-7000, Hitachi,Tokyo, Japan) was used to determine the three-dimensional excitation-emission matrix (EEM) of the fluorescent organic components in SMP and EPS in each tank of three bioreactors. The operational parameters were set according to Maqbool et al. [1]. Excitation-emission matrix-parallel factor (EEM-PARAFAC) analysis was introduced to elucidate the fluorescence peaks of different compounds [2]. A dataset contained 420 EEMs of SMP and EPS from three bioreactors was used for PARAFAC modeling. A free DOMFlour toolbox of Matlab was used and the detailed analysis procedures could be found elsewhere [3]. The maximum fluorescence intensities (F_{max}) of individual components were in direct proportion to their relative concentrations [4].

Table S1. Chemical composition of synthetic wastewater.

Components	Concentration (mg/L)	
	Control	AOA
Glucose	1000	1185
NH_4Cl	140	165.9
KH_2PO_4	25	29.63
$NaNO_3$	40	47.4
$MgSO_4.7H_2O$	5	5.93
$FeCl_3$	2.33	2.76
$NaHCO_3$	Appropriate to maintain pH = 7.0	Appropriate to maintain pH = 7.0

Table S2. The average ± standard deviation of basic quality parameters of influent and effluent in three bioreactors.

Items	Influent (mg/L)		Effluent (mg/L)			Removal Efficiency（%）		
	$MBR_{control}$	AOA	$MBR_{control}$	AOA_{S150}	AOA_{S300}	$MBR_{control}$	AOA_{S150}	AOA_{S300}
TOC	389.98± 5.94	441.2 ± 10.64	3.62±0.48	4.87 ± 1.55	4.8 ± 1.56	> 99	> 98	> 98
TN	45.20±2.72	52.88±6.30	8.49 ± 3.27	2.82 ± 1.17	3.59 ± 1.76	> 81	> 94	>93
TP	6.13±0.07	7.34 ± 0.19	1.16 ± 0.56	1.70 ± 0.49	1.60 ± 0.57	> 81	> 76	> 78

Table S3. Sequencing of bacterial 16S rRNA gene along with alpha diversity of microbial taxa in three bioreactors.

Sample description	Sequencing results		Species diversity
	Effective tags	OTUs	Simpson
$MBR_{control}$-A-1d-a_1	56402	1720	0.018
$MBR_{control}$-O-1d-o_1	65627	1449	0.11
$MBR_{control}$-M-1d-m_1	58914	1520	0.116
$MBR_{control}$-A-45d-a_2	58448	1627	0.057
$MBR_{control}$-O-45d-o_2	67622	1489	0.0694
$MBR_{control}$-M-45d-m_2	56745	1268	0.0544
$MBR_{control}$-A-90d-a_3	61203	1232	0.133
$MBR_{control}$-O-90d-o_3	59066	1225	0.114
$MBR_{control}$-M-90d-m_3	61120	1258	0.115
AOA_{S150}-A-1d-a_4	59282	1395	0.064
AOA_{S150}-O-1d-o_4	63154	1195	0.0953
AOA_{S150}-M-1d-m_4	60434	1370	0.0596
AOA_{S150}-A-45d-a_5	63338	1080	0.18
AOA_{S150}-O-45d-o_5	68505	1039	0.29
AOA_{S150}-M-45d-m_5	64844	1194	0.211
AOA_{S150}-A-90d-a_6	50333	1236	0.0588
AOA_{S150}-O-90d-o_6	61612	1185	0.0836
AOA_{S150}-M-90d-m_6	62850	1186	0.0913
AOA_{S300}-A-1d-a_7	58914	1520	0.094
AOA_{S300}-O-1d-o_7	59902	1288	0.0568
AOA_{S300}-M-1d-m_7	59713	1575	0.0472
AOA_{S300}-A-45d-a_8	56745	1268	0.0784
AOA_{S300}-O-45d-o_8	68153	1121	0.0924
AOA_{S300}-M-45d-m_8	58512	1169	0.12
AOA_{S300}-A-90d-a_9	61120	1258	0.11
AOA_{S300}-O-90d-o_9	57553	1240	0.115
AOA_{S300}-M-90d-m_9	58938	1132	0.123

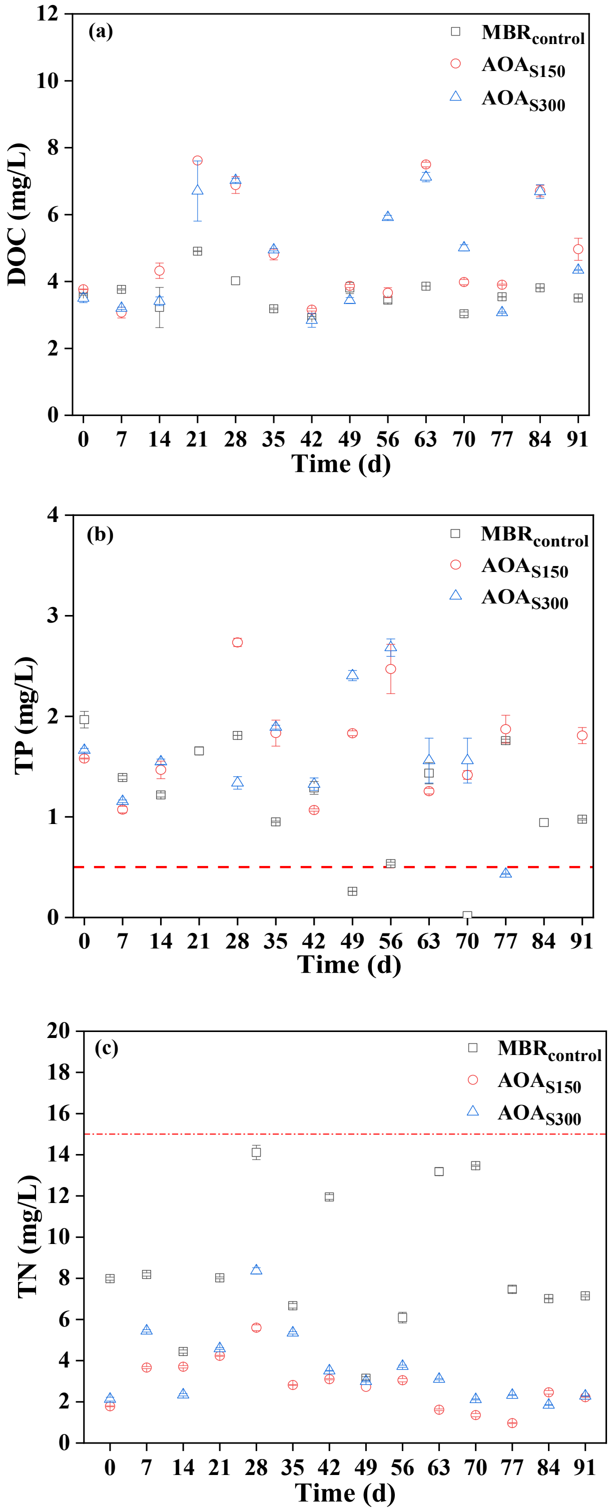

Figure S1. The temporal variations in basic quality parameters (a) DOC, (b) TN, and (c) TP in effluents from three bioreactors.

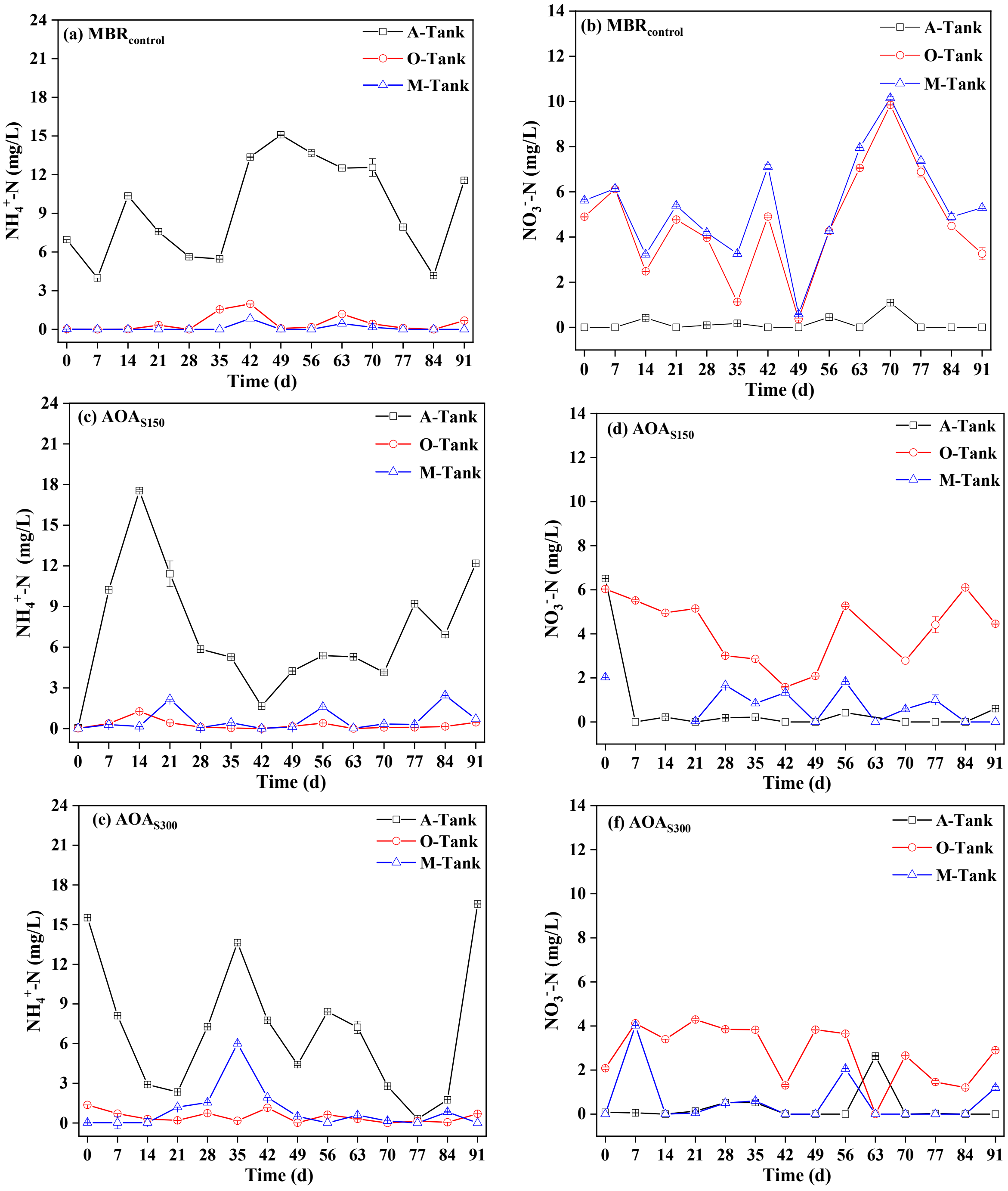

Figure S2. The temporal variations in NH_4^+-N and NO_3^--N concentrations in different tanks of three bioreactors.

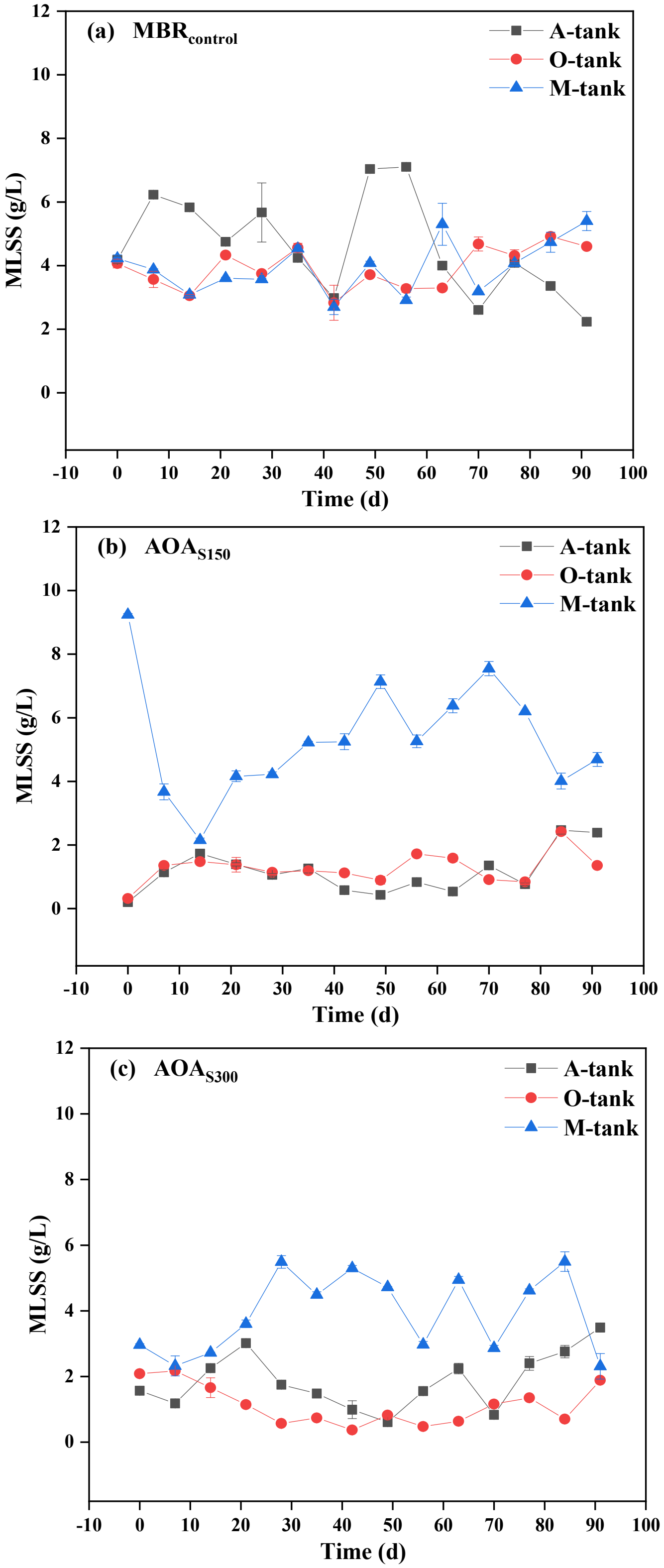

Figure S3. The changes of MLSS concentration in different tanks of three bioreactors.

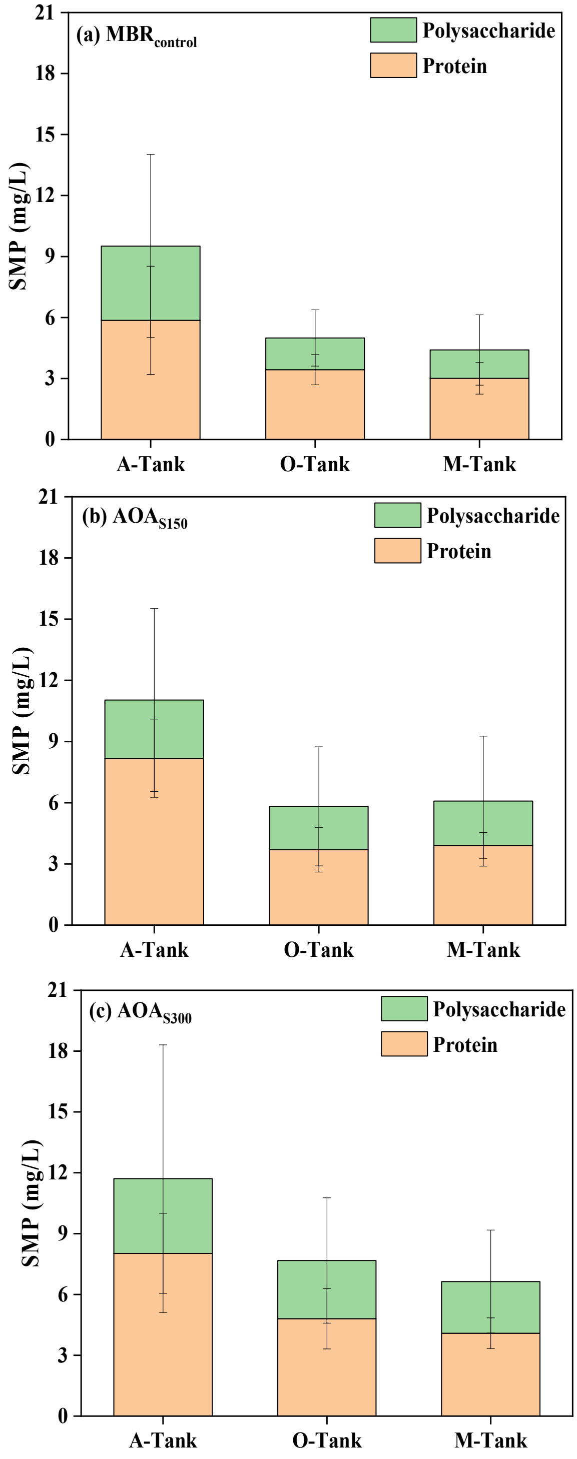

Figure S4. The average polysaccharide and protein in SMP in different tanks of MBR$_{control}$ (a), AOA$_{S150}$ (b), and AOA$_{S300}$ (c).

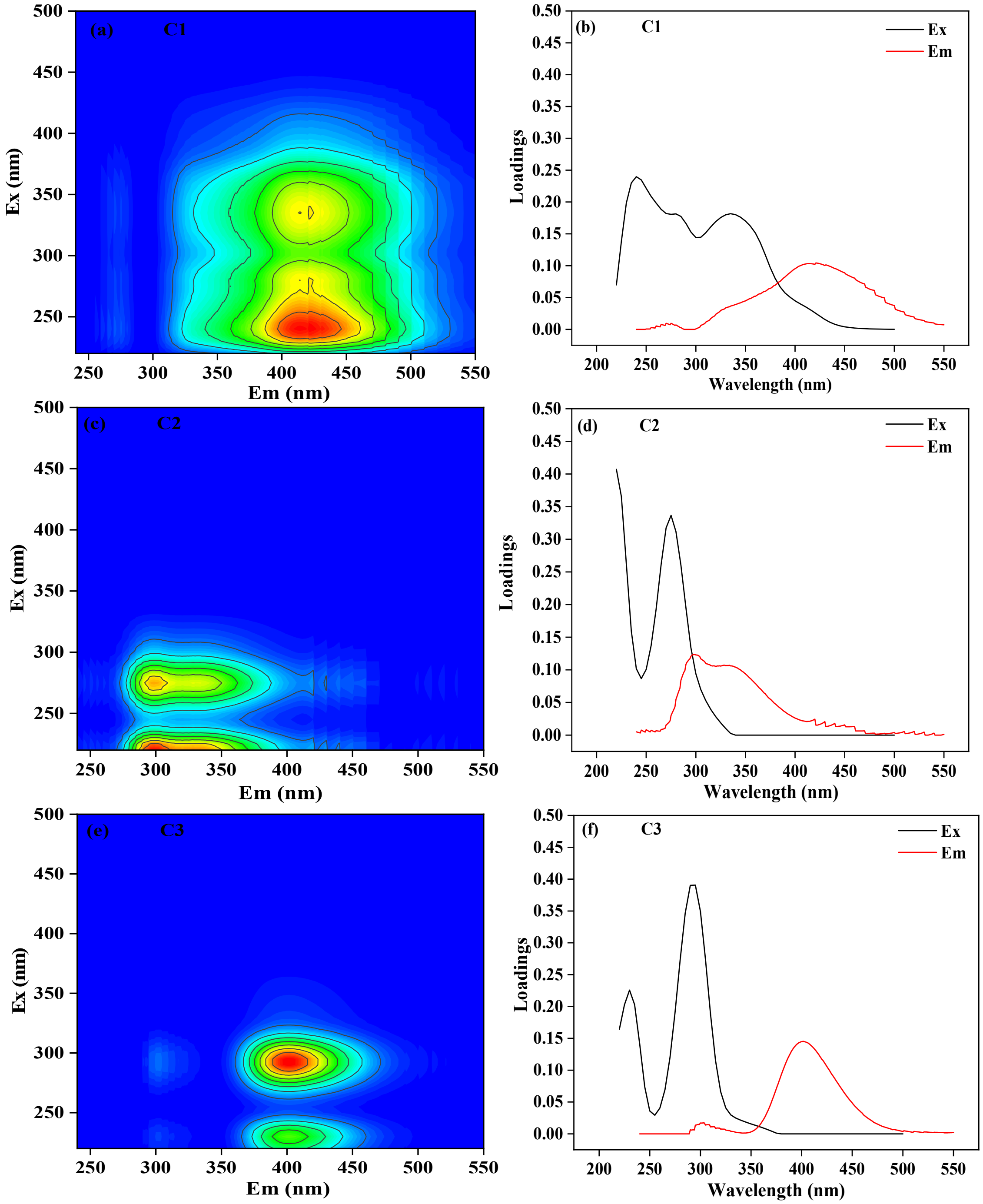

Figure S5. EEM-PARAFAC components, humic-like (C1), tryptophan-like (C2), and fulvic-like (C3).

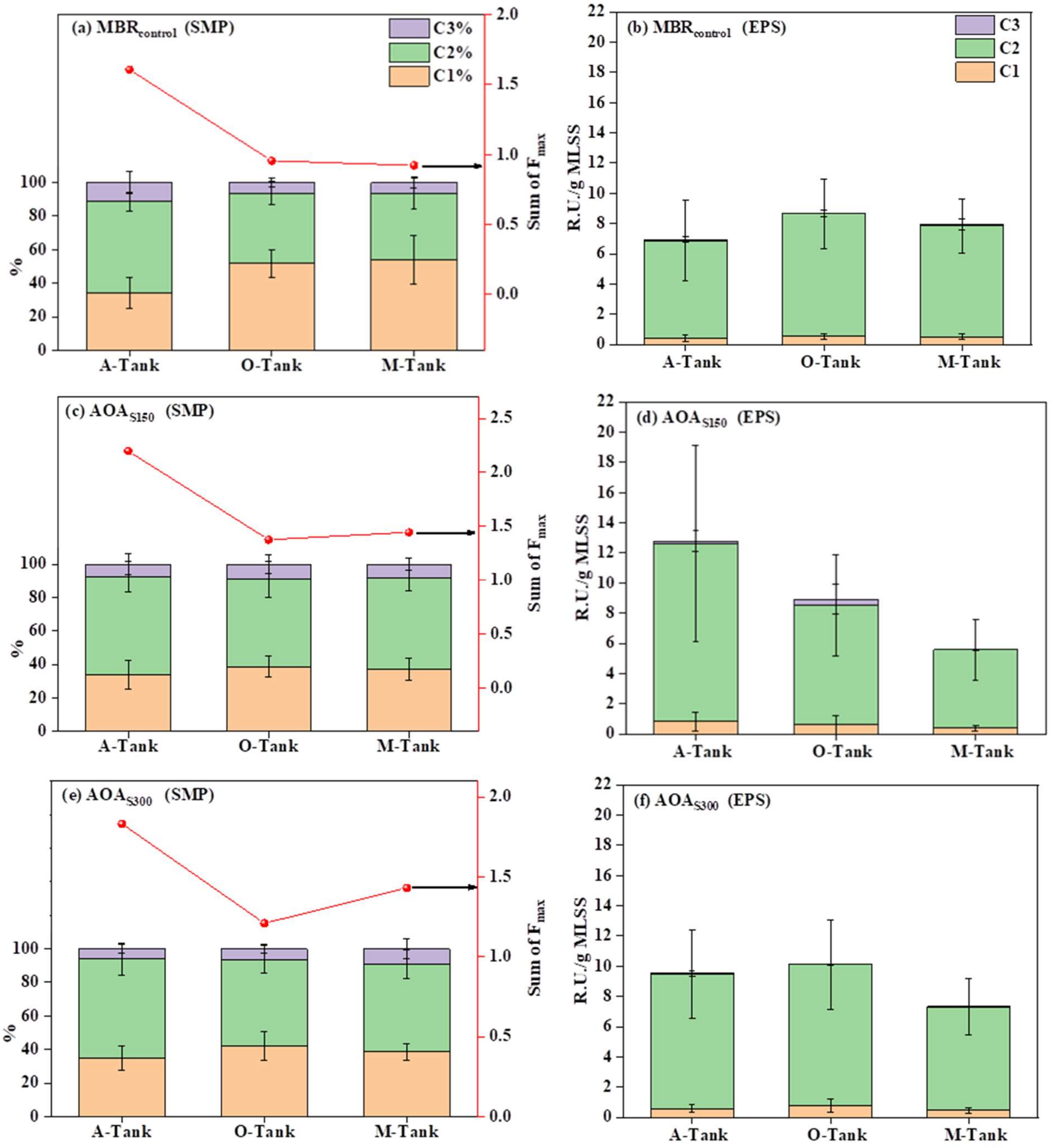

Figure S6. The average distribution of EEM-PARAFAC components and sum of F_{max} (C1+C2+C3) in SMP and EPS of different tanks in $MBR_{control}$ (a and b), AOA_{S150} (c and d), and AOA_{S300} (e and f).

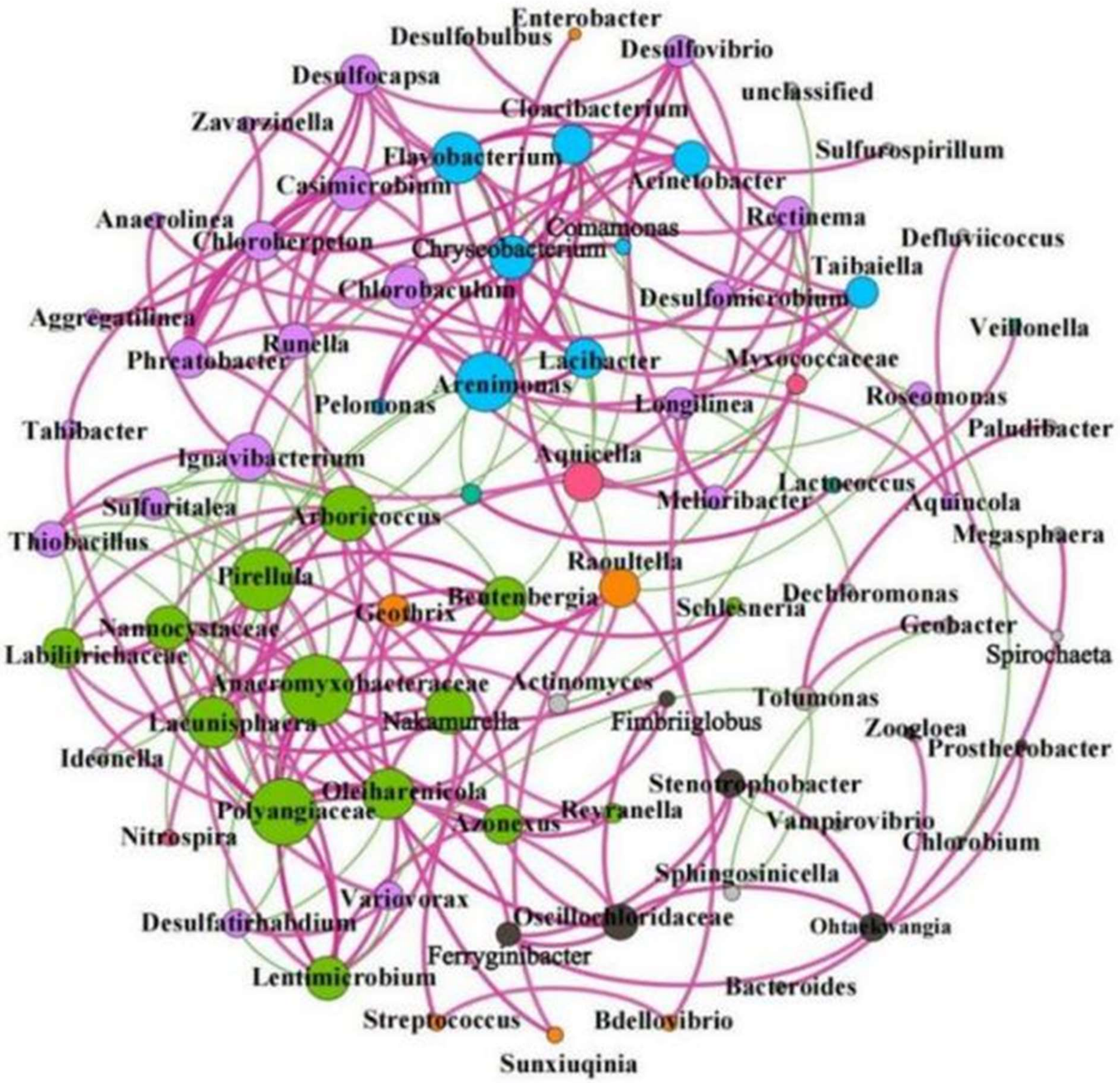

Figure S7. The network analysis among functional microorganisms in three bioreactors.

References

1. Maqbool, T., Li, C., Qin, Y., Zhang, J., Asif, M. B., Zhang, Z. . A year-long cyclic pattern of dissolved organic matter in the tap water of a metropolitan city revealed by fluorescence spectroscopy. *Sci. Total Environ.* **2021**, 771, 144850. https://doi.org/10.1016/j.scitotenv.2020.144850

2. Huang, M., Li, Z., Huang, B., Luo, N., Zhang, Q., Zhai, X., Zeng, G. Investigating binding characteristics of cadmium and copper to DOM derived from compost and rice straw using EEM-PARAFAC combined with two-dimensional FTIR correlation analyses. *J. Hazard. Mater.* **2018**, 344, 539-548. https://doi.org/10.1016/j.jhazmat.2017.10.022

3. Stedmon, C. A., Bro, R. Characterizing dissolved organic matter fluorescence with parallel factor analysis: a tutorial. Limnol. Oceanogr.-Meth. **2008**, 6(11), 572-579. https://doi.org/10.4319/lom.2008.6.572

4. Rodríguez-Vidal, F. J., García-Valverde, M., Ortega-Azabache, B., González-Martínez, Á., Bellido-Fernández, A. Characterization of urban and industrial wastewaters using excitation-emission matrix (EEM) fluorescence: Searching for specific fingerprints. *J. Environ. Manage.* **2020**, 263, 110396. https://doi.org/10.1016/j.jenvman.2020.110396
